# Combination of quercetin and 2-methoxyestradiol inhibits epithelial–mesenchymal transition in PC-3 cell line via Wnt signaling pathway

**DOI:** 10.2144/fsoa-2021-0028

**Published:** 2021-09-24

**Authors:** Neeti Sharma, Piyush W Raut, Meghna M Baruah, Akshay Sharma

**Affiliations:** 1 School of Engineering, Ajeenkya DY Patil University, Charholi Budruk, Pune, 412105, India; 2 Symbiosis School of Biological Sciences, Symbiosis International (Deemed University), Gram – Lavale; Taluka – Mulshi, Pune, India

**Keywords:** 2-methoxyestradiol, EMT, metastasis, prostate cancer, quercetin, Wnt signaling

## Abstract

**Aim:**

We have previously reported that quercetin (Qu) regulates epithelial–mesenchymal transition (EMT) by modulating Wnt signaling components. In this study, we investigated the synergistic effect of Qu and 2-methoxyestradiol (2-ME) and the role of Wnt signaling components in regulating EMT in PC-3 cells.

**Materials & methods:**

EMT was induced by treating PC-3 cells with TGF-β, followed by evaluation of expression of EMT markers and Wnt signaling proteins in naive, induced and after exposing induced cells to Qu and 2-ME at both gene and protein level by real-time PCR (RT-PCR) and western blot, respectively.

**Results:**

Qu and 2-ME synergistically downregulated mesenchymal markers with simultaneous upregulation of epithelial markers. Wnt signaling proteins expression was also downregulated by Qu and 2-ME in TGF-β-induced EMT in PC-3 cells.

**Conclusion:**

Thus, combination therapy of Qu and 2-ME could be a new promising therapeutic approach for the treatment of prostate cancer.

## Lay abstract

The current study describes the synergistic effect of quercetin and 2-methoxyestradiol and the role of Wnt signaling components in regulating epithelial–mesenchymal transition (EMT) in PC-3 cells. EMT was induced by treating PC-3 cells with TGF-β, followed by the evaluation of expression of EMT markers and Wnt signaling proteins in naive and induced states. Quercetin and 2-methoxyestradiol could synergistically downregulate mesenchymal markers with simultaneous upregulation of epithelial markers along with the downregulation of Wnt signaling proteins.

Prostate cancer (PCa) is one of the leading causes of cancer deaths among men, with an estimated number of 164,690 new cases and 29,430 deaths in the USA in 2018 [1,2]. Metastasis is a complex, multistep process wherein tumor cells acquire mesenchymal characteristics to escape their original site and propagate to distant sites [3]. Epithelial to mesenchymal transition (EMT) is a plastic transition that aids in tumor progression by imparting invasive phenotype to tumors of epithelial origin [4]. These epithelial cells gain mesenchymal properties with increased N-cadherin expression and decreased E-cadherin expression, thus leading to invasiveness.

EMT is stimulated by various signals from the tumor microenvironment, which includes various factors and cytokines. Members of the TGF-β superfamily are potent inducers of EMT [5]. TGF-β is known to exhibit opposite roles during PCa progression, acting both as a tumor suppressor or promoter during the early and later stages, respectively. In the benign stages of PCa, TGF-β induces apoptosis after binding to its receptors. Besides that, it regulates cellular processes such as differentiation, proliferation as well as migration [5]. In the late stages of PCa, upregulated TGF-β has been known to increase cell invasion and metastasis [6]. It also regulates EMT by downregulation of epithelial markers *viz.*, E-cadherin and upregulation of mesenchymal markers such as vimentin [7]. Therefore, targeting the EMT process may have important therapeutic implications in the cancer management [8].

The EMT process is tightly controlled in a complex manner by various signaling pathways such as SMAD, PI3K/Akt, MAPK, mTOR, Ras and Wnt, among others [8]. Differential expression of Wnt has been associated with the aggressive form of PCa [9]. The high mutation rates in the Wnt signaling components as observed in many different cancers as well as their association with initiation and progression in various cancers reveal their role in carcinogenesis [10]. Thus, studying the molecular mechanisms underlying the association of Wnt activation and PCa aggressiveness may prove to be an essential step toward the cure for metastatic PCa [11].

Cancer chemoprevention, by using natural or dietary agents, is increasingly becoming popular to combat increasing cases of cancers. Several studies have emphasized the potential benefits of flavonoids for cancer prevention. Quercetin (Qu) is one such flavonoid that has been reported to inhibit the growth of cancer cells in several tumors such as breast cancer, cervical cancer, colon, lung carcinoma and PCa [11–16] and is nontoxic. Various *in vitro* and *in vivo* studies on PCa have demonstrated that Qu alone or in combination with some other drug effectively inhibits angiogenesis, inhibits proliferation, impairs cell viability, reduces cell migration, induces apoptosis and arrests cell cycle [17,18]. In plants, Qu exists as hydrophilic glycosides, lowering its bioavailability [19]. Low bioavailability of Qu limits its application in clinical setups and thus, research focuses on using Qu in synergy with other anticancer drugs besides modification of its molecular structure.

In the last decade, 2-methoxyestradiol (2-ME) has gained significant interest due to its antiapoptotic, antiproliferative, antiangiogenic activities in many cancers, including PCa [20]. In PCa, it is known to inhibit tumors in both androgen-independent and androgen-dependent PCa both *in vitro* and *in vivo* [21–23]. However, 2-ME is associated with limited bioavailability and rapid degradation [24], and thus combined drug therapy has attracted the interest of many researchers.

Recently, it has been shown that Qu and 2-ME synergistically exhibit an anticancer effect on both androgen-independent and androgen-dependent PCa cells at both *in vitro* and *in vivo* levels [14,25]. However, the underlying molecular mechanisms and the signaling pathway regulated by these drugs are not well studied.

Previous work from our laboratory has shown the potential of Qu in modulating Wnt signaling in TGF-β-induced EMT in PC-3 cells [11]. Our work revealed that Qu reduced TGF-β-induced expression of vimentin and N-cadherin and increased E-cadherin expression in PC-3 cells, thus preventing TGF-β-induced EMT. Furthermore, it was reported that Qu significantly decreased the TGF-β-induced expression of Twist, Snail and Slug. Based on the above pieces of evidence, we were encouraged to investigate the role of Qu and 2-ME combinations on TGF-β-induced EMT in PC-3 cells. In the present study, we evaluated the molecular signaling pathway being exploited by Qu and 2-ME alone and in synergy to exert their anticancer properties on PCa cells with the regulation of EMT markers. We have also shed light on the antioxidant properties of these flavonoids individually and in synergy and on the synergistic effect of these compounds on the metastatic potential of PCa cells (PC-3).

## Materials & methods

### Cell culture

PC-3 cells were obtained from National Center for Cell Science, Pune, India. The cells were grown in Rosewell Park Memorial Institute Medium (RPMI-1640), containing 10% fetal bovine serum and supplemented with 100 U/ml penicillin and l00 μg/ml streptomycin antibiotic solution, and were maintained in a humidified incubator with 95% air and 5% CO_2_ at 37°C. The cells were trypsinized using 1× trypsin EDTA on reaching 80–90% confluency.

### Drug treatment

Qu and 2-ME were dissolved in DMSO at 20 and 10 μM, respectively. Qu concentration of DMSO was added to media to be used as vehicle control. TGF-β (10 ng/ml) was used to induce EMT in PC-3 cells. Drug treatment was given in three groups *viz.*, group 1: Qu (20 μM); group 2: 2-ME (10 μM) and group 3: combination (Qu + 2-ME).

### Cell viability assay

PC-3 cells were seeded in a 96-well plate at a density of 1 × 10^4^ cells/well in 200 μl of RPMI containing 10% fetal bovine serum and incubated for 24 h in humidified CO_2_ incubator at 37°C. After 24 h of seeding the cells, the old media were replaced with growth media containing Qu and 2-ME at concentrations 20 and 10 μM, respectively. The cells were then allowed to incubate for 24–72 h in humidified CO_2_ incubator at 37°C. To check the cell viability, 50 μl of MTT (3–4,5-diamethylthiazol-2-yl-2,5-diphenyltetrazolium bromide) working solution in phosphate-buffered saline was prepared at a concentration of 1 mg/ml, was added to each well and was incubated for 4 h. After 4 h of incubation, the formazon complexes formed were dissolved in 150 μl of acidic isopropanol. The colorimetric estimation of the formazon complex was done every 24 h of treatment at 570 nm using an ELISA plate reader. A graph was plotted with Qu and 2-ME concentration on X-axis and % proliferation on Y-axis.

### Cell migration assay

PC-3 cells were seeded in a 24-well plate at a density of 2 × 10^4^ cells/well and were incubated to form a monolayer at 37°C in a humidified CO_2_ incubator. After formation of a confluent monolayer, the old media were removed and the cells were scraped with a sterile 200 μl tip to form a ‘scratch’. Then, 1-ml complete growth media containing additives such as TGF-β (10 ng/ml), Qu (20 μM), Qu (20 μM) + TGF-β (10 ng/ml), 2-ME (10 μM), 2-ME (10 μM) + TGF-β (10 ng/ml), Qu (20 μM) + 2-ME (10 μM) and Qu (20 μM) + 2-ME (10 μM) + TGF-β (10 ng/ml), were added to each well. Pictures were clicked every 24 h using an inverted microscope to observe the sealing of the gap with different treatment conditions.

### Colony formation assay

PC-3 cells were seeded at a density of 5 × 10^4^ in 6-well plates and were introduced to drug treatment as mentioned above. Soft agar plates were prepared using 1% base agar and 0.7% top agar layers. After 24 h of treatment, cells were harvested and suspended in a serum-free medium and plated in soft agar plates. Pictures were taken every 24 h to observe the transformation of cells.

### Cell cycle distribution

Cell cycle analysis was performed by flow cytometry using propidium iodide (PI) staining. PC-3 cells were seeded at concentration 2 × 10^6^ cells and serum-starved for 4 h followed by drug treatment. Cells were harvested, and the percentage of cells in different phases of the cell cycle was analyzed using BD FACS Calibur.

### Apoptosis detection

Caspase 3 assay kit (Sigma-Aldrich, MO, USA) was used to detect caspase 3 activity. PC-3 cells were harvested after 24 h of drug treatment at a concentration of 2 × 10^6^ cells. The cell lysate was prepared using 1× cell lysis buffer provided with the kit. The concentration of *p*-nitroaniline released from each treated sample compared with the untreated samples was calculated from the absorbance value at 405 nm.

### Alkaline phosphatase activity

The alkaline phosphatase (ALP) activity of the PC-3 cells after drug treatment was examined by EZAssay Alkaline Phosphatase Activity Estimation Kit (HiMedia, Mumbai, India). PC-3 cells were seeded in 60 mm dishes at a concentration of 5 × 10^5^ cells and protein lysate was prepared using 1× cell lysis buffer. ALP activity was measured at 405 nm according to the manufacturer’s instructions.

### Reactive oxygen species analysis

Reactive oxygen species (ROS) activity of PC-3 cells after drug treatments was assessed by Griess assay. PC-3 cells were seeded at a density of 5 × 10^5^ cells in a 24-well plate. After 24 h of drug treatment, 50 μl of 1% sulfanilamide solution was added to each well and was incubated for 5–10 min at room temperature in dark. Then, 50 μl of 0.1% *N*-1-napthylethylenediamine dihydrochloride was added to each well and was incubated 5–10 min at room temperature in dark. The absorbance of the purple-colored complex thus formed was measured at 540 nm.

### Isolation of total cellular RNA & real-time PCR

Total RNA was isolated by Trizol reagent (Sigma). The isolated RNA was quantified using NanoDrop UV Spectrophotometer. Two microgram of total cellular RNA was reverse transcribed using High Capacity cDNA Synthesis Reverse Transcription Kit (Applied Biosystems, MA, USA). Real-time (RT)-PCR was performed in Applied Biosystems StepOne Plus using SYBR green, and the data were normalized using β-actin as endogenous control. The fold change of the target genes were analyzed using the comparative CT method.

### Western blot

PC-3 cells were harvested after 24 h of drug treatment. 1× Radioimmunoprecipitation assay buffer with 1× protease inhibitor cocktail was used to lyse the cells. The cell lysate thus obtained was centrifuged at 10,000 rpm at 4°C for 15 min to remove the cell debris and the supernatant obtained was stored at -80°C. The protein samples were estimated by Bradford assay. Equal amount of protein samples (30 μg) were separated using 12% SDS-PAGE gels and transferred to nitrocellulose membrane. The membrane was then incubated overnight with primary antibodies against CyclinD1 (1:500 dilution), β-catenin (1:200 dilution), GSK-3β (1:500 dilution) in 4°C. The membrane was then incubated with horseradish peroxidase-conjugated secondary anti-mouse IgG antibody (1:5000 dilution) or horseradish peroxidase-conjugated secondary antirabbit IgG antibody (1:5000 dilutions) for 1 h at room temperature. Gel doc (Diversity 4, Syngene, India) was used to visualize the chemiluminescence.

### Statistical analysis

All experiments were performed in triplicates. Data were presented as mean ± SEM. Statistical analysis was performed using ANOVA with Tukey’s *post hoc* multicomparison test in GraphPad Prism version 5. p < 0.05 was considered to be statistically significant. The significance levels were defined as *p < 0.05; **p < 0.01; ***p < 0.001.

## Results

### The combination of Qu & 2-ME decreased the viability of PC-3 cells

**Figure 1.**
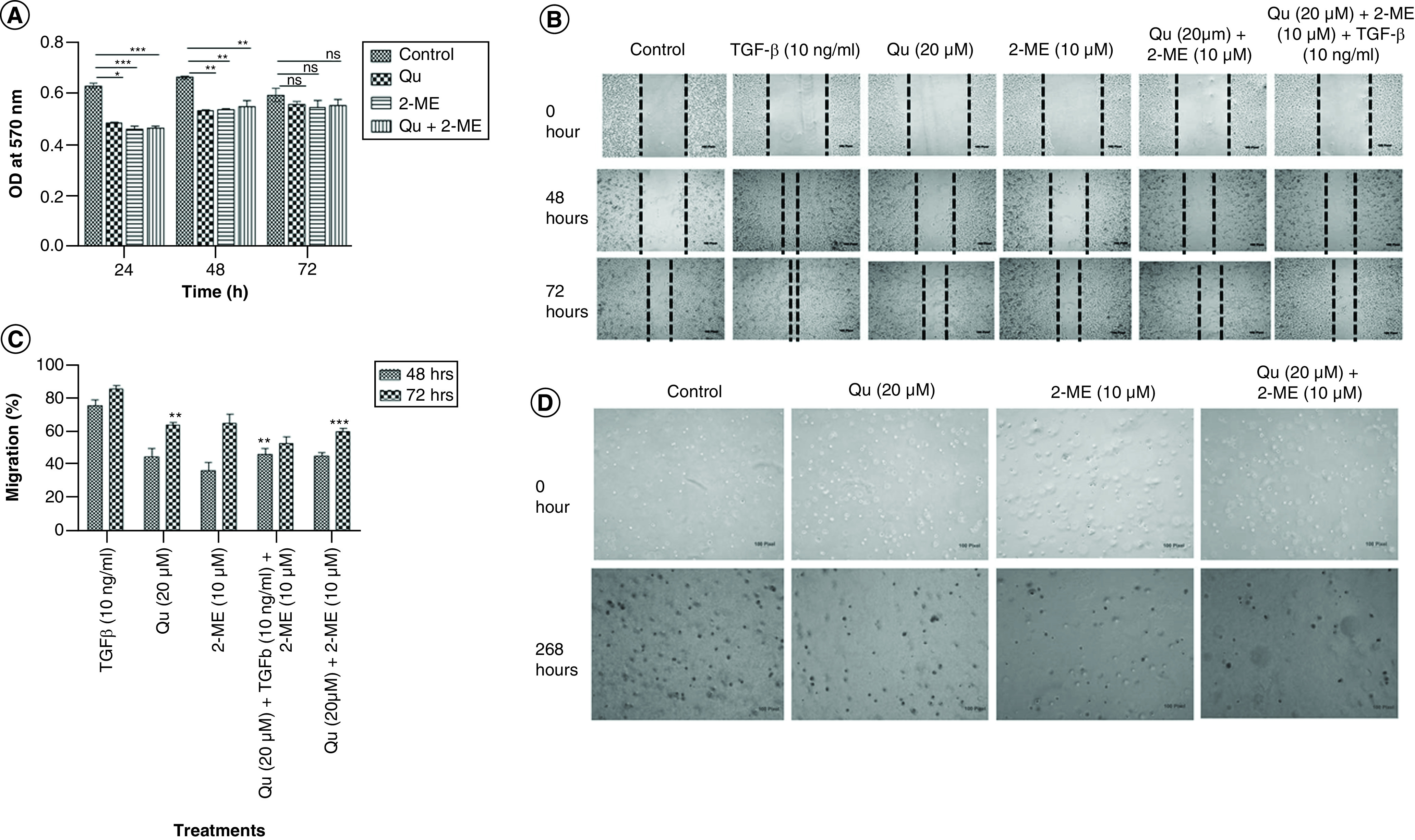
Quercetin and 2-methoxyestradiol inhibit cell proliferation in a time-dependent manner with simultaneous synergistic inhibition of TGF-β-induced migration and colony-forming ability of PC-3 cells. **(A)** Effect of drug treatment on PC-3 cell proliferation was analyzed by MTT assay, normalized with the control group and graph was plotted with OD at 540 nm on Y-axis and time on X-axis. Data represented as mean ± SEM from three independent experiments. The statistical analysis was performed using ANOVA with Tukey’s *post hoc* multicomparison test (*p < 0.05; **p < 0.01; ***p < 0.001). **(B)** Cell migration in scratch assay after treatment with all the three groups of drugs. Pictures were taken using an inverted microscope in various time points in triplicates. Data represented as mean ± SEM from three independent experiments. **(C)** Histogram plots with the percentage migration in the respective treatment group are shown as mean ± SEM from three independent experiments. The statistical analysis was performed using ANOVA with Tukey’s *post hoc* multicomparison test (*p < 0.05; **p < 0.01; ***p < 0.001). **(D)** Soft agar assay was performed to determine the colony-forming ability of PC-3 cells. PC-3 cells were harvested after 24 h of treatment and suspended in serum-free medium and plated in soft agar dishes prepared using 1% base agar and 0.7% top agar layers. Pictures were taken every 24 h using an inverted microscope to observe the transformation of cells. 2-ME: 2-methoxyestradiol; EMT: Epithelial–mesenchymal transition; OD: Optical density; Qu: Quercetin.

The inhibitory effect of Qu and 2-ME on the proliferation of PC-3 cells at concentrations 20 and 10 μM was determined using MTT assay as explained in the ‘Materials & methods’ section. As shown in Figure 1A, both Qu and 2-ME alone were found to inhibit proliferation of PC-3 cells in a time-dependent manner. However, a significant higher rate of inhibition was observed in the combination group compared with the individual groups.

### The combination of Qu & 2-ME decreased the migration of PC-3 cells

Wound-healing assay (scratch assay) was performed to study the migration of PC-3 cells. It was observed that the migratory potential of PC-3 cells was reduced upon treatment with Qu (20 μM), 2-ME (10 μM) and Qu (20 μM) + 2-ME (10 μM), however, treatment with Qu (20 μM) + 2-ME (10 μM) showed an enhanced inhibitory effect highlighting their synergistic role in inhibiting migration of PC-3 cells (Figure 1B). Percentage migration graph was plotted against the treatment conditions with respect to control wells and it was observed that treatment with TGF-β (10 ng/ml) showed the highest migration of cells and treatment with Qu (20 μM) and 2-ME (10 μM) could inhibit the same in a time-dependent manner (Figure 1C). Moreover, cells treated with the combination group of drugs showed the lowest % migration indicating their synergistic effect on inhibition of migration of PC-3 cells.

### The combination of Qu & 2-ME decreased the colony-forming ability of PC-3 cells

Cancer cells have the property of forming spheres in serum-free media or soft agar. Treatment with Qu (20 μM), 2-ME (10 μM) and Qu (20 μM) + 2-ME (10 μM) inhibited the colony-forming ability of PC-3 cells, and a higher rate of inhibition was observed in cells treated with Qu (20 μM) + 2-ME (10 μM) compared with untreated cells (Figure 1D).

### The combination of Qu & 2-ME led to sub-G0/G1 arrest of PC3 cells

**Figure 2.**
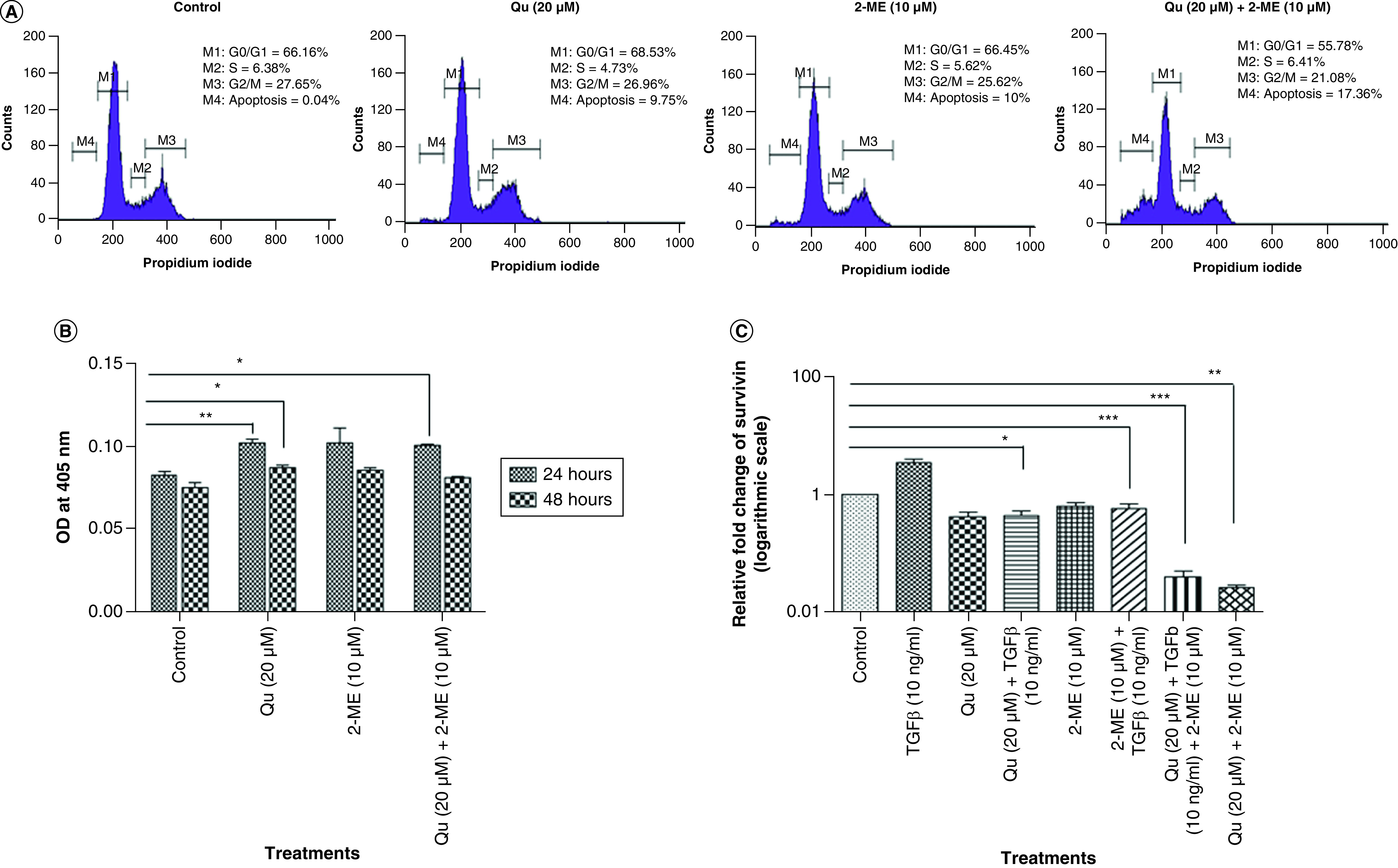
Quercetin and 2-methoxyestradiol synergistically induced apoptosis in PC-3 cells by arresting cells in sub-G0/G1 phase and exhibiting enhanced caspase-3 activity by downregulating inhibitor of apoptosis protein. **(A)** Graph depicting the percentage of cells in different phases of the cell cycle was analyzed after treatment with all the three groups of drug. **(B)** Quantization of cleaved caspase 3 in PC-3 cells after treatment with all the three treatment groups. Data represented as mean ± SEM from three independent experiments. The statistical analysis was performed using ANOVA with Tukey’s *post hoc* multicomparison test (*p < 0.05; **p < 0.01; ***p < 0.001). **(C)** Effect of all the three different groups of drug on the mRNA expression of survivin. Gene levels were normalized with β-actin and expressed as fold change with respect to the control group. Data represented as mean ± SEM from three independent experiments. The statistical analysis was performed using ANOVA with Tukey’s *post hoc* multicomparison test (*p < 0.05; **p < 0.01; ***p < 0.001). 2-ME: 2-methoxyestradiol; EMT: Epithelial–mesenchymal transition; OD: Optical density; Qu: Quercetin.

To evaluate the effect of drug treatment on cell-cycle distribution of PC-3 cells, flow cytometry analysis was performed using PI staining. Treatment with Qu and 2-ME individually caused G0/G1 arrest, whereas the combination group showed sub-G0/G1 arrest indicating the synergistic effect of Qu and 2-ME in inducing apoptosis of PC-3 cells (Figure 2A).

### Qu & 2-ME combination led to an increase in apoptosis of PC3 cells

Caspase 3 activity was measured to determine the effect of drug treatment on apoptosis of PC-3 cells. Treatment with Qu (20 μM), 2-ME (10 μM) and Qu (20 μM) + 2-ME (10 μM) could significantly increase caspase 3 activity of PC-3 cells (Figure 2B), indicating induction of apoptosis upon drug treatment on PC-3 cells.

### Qu & 2-ME combination led to downregulation of inhibitor of apoptosis family proteins in PC3 cells

The expression level of survivin (BIRC5), a known inhibitor of apoptosis and regulator of transformed cells [26], was studied by RT-PCR. It was observed that TGF-β treatment significantly upregulated the expression of survivin, whereas treatment with Qu (20 μM), 2-ME (10 μM) and Qu (20 μM) + 2-ME (10 μM) could downregulate the same (Figure 2C). The maximum downregulation was observed in the group treated with a combination drug followed by treatment with Qu (20 μM) and 2-ME (10 μM) alone.

### Synergistic decrease in ALP activity of PC-3 cells by Qu & 2-ME

**Figure 3.**
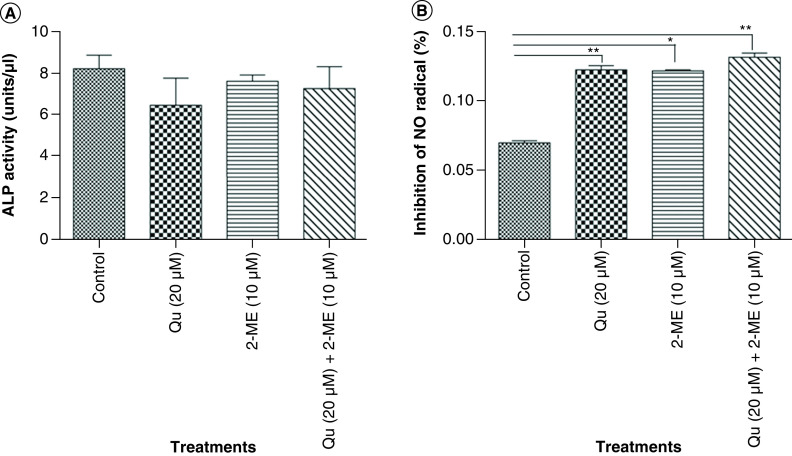
Alkaline phosphatase and reactive oxygen species activity of PC-3 cells. **(A)** Depicts % inhibition of NO_2_ upon treatment with all the three groups of drug. Data represented as mean ± SEM from three independent experiments. The statistical analysis was performed using ANOVA with Tukey’s *post hoc* multicomparison test (*p < 0.05; **p < 0.01; ***p < 0.001). **(B)** ALP activity was analyzed using colorimetric alkaline phosphates activity detection kit. Data represented as mean ± SEM from three independent experiments. The statistical analysis was performed using ANOVA with Tukey’s *post hoc* multicomparison test (*p < 0.05; **p < 0.01; ***p < 0.001). 2-ME: 2-methoxyestradiol; ALP: Alkaline phosphatase; EMT: Epithelial–mesenchymal transition; Qu: Quercetin.

ALP activity was assessed to determine the effect of drug treatment on the metastatic potential of PC-3 cells. ALP is a known marker of metastasis in PCa cells to bone [27,28]. Our results showed that all the three groups of drug treatment could inhibit the expression level of ALP with the highest inhibition was observed in the cells treated with Qu (20 μM) alone (6.50 units/ml), followed by combination group (7.30 units/ml) and 2-ME (10 μM) (7.65 units/ml) (Figure 3A).

### Qu & 2-ME combination caused an increase in ROS activity of PC3 cells

ROS activity was measured using Griess assay as explained in the ‘Materials & methods’ section. ROS activity of PC-3 cells was found to increase upon drug treatment compared with untreated cells (Figure 3B).

### The combination of Qu & 2-ME decreased the expression of mesenchymal markers

**Figure 4.**
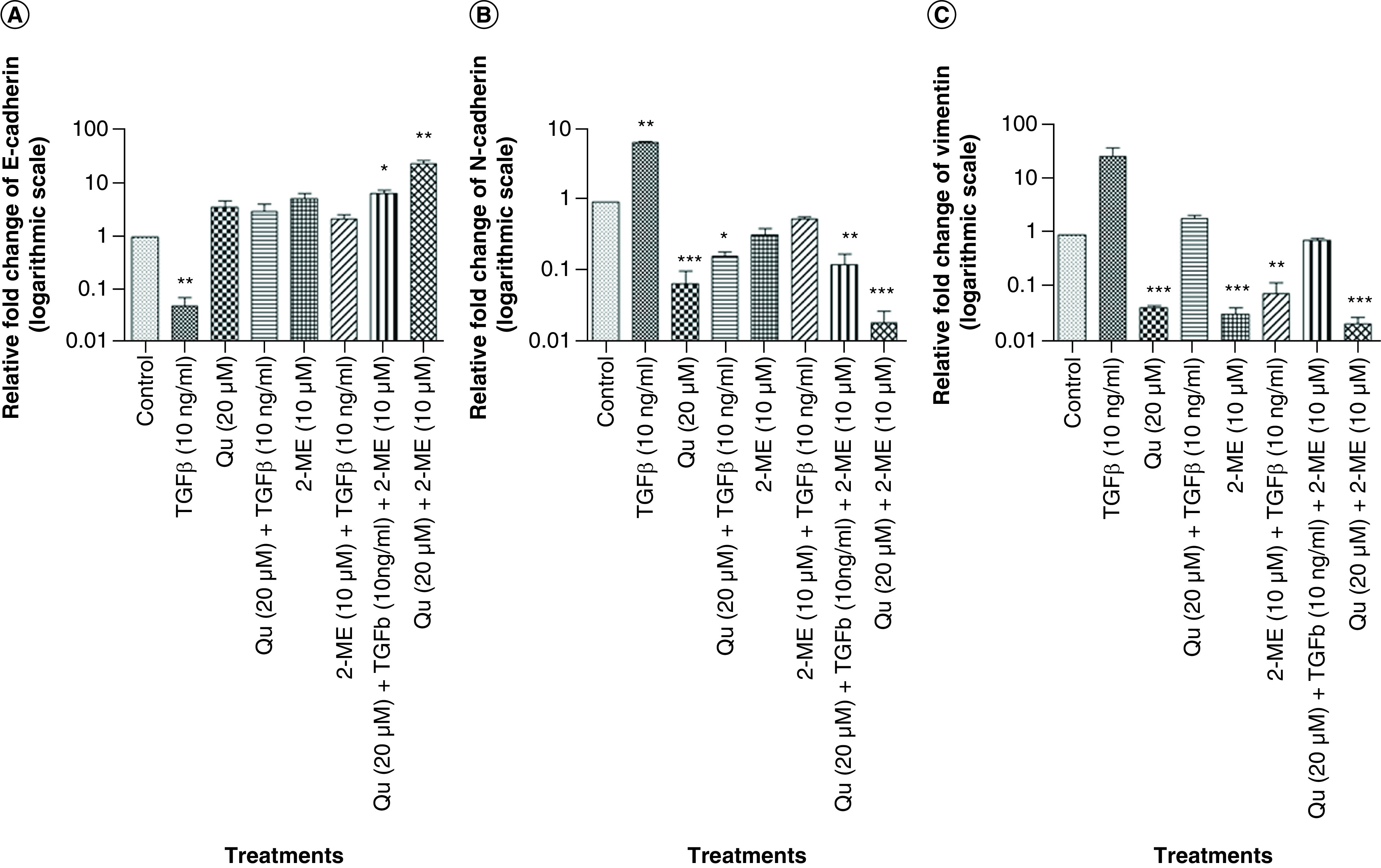
Combination of quercetin and 2-methoxyestradiol inhibited epithelial–mesenchymal transition in PC-3 cells by downregulating the expression of mesenchymal markers and upregulation of epithelial markers. Effect of all the three different groups of drugs on the mRNA expression of EMT markers. **(A)** E-cadherin. **(B)** N-cadherin. **(C)** Vimentin. Gene mRNA levels were normalized with β-actin and expressed as fold change with respect to the control group. Data represented as mean ± SEM from three independent experiments. The statistical analysis was performed using ANOVA with Tukey’s *post hoc* multicomparison test (*p < 0.05; **p < 0.01; ***p < 0.001). 2-ME: 2-methoxyestradiol; EMT: Epithelial–mesenchymal transition; Qu: Quercetin.

The expression level of the characteristic markers of EMT *viz.*, E-cadherin, N-cadherin and vimentin were evaluated by RT-PCR to determine the effectiveness of the drug treatment on EMT. Treatment with TGF-β downregulated E-cadherin expression with simultaneous upregulation of mesenchymal markers (N-cadherin and vimentin). However, upon treatment with all three groups of drugs, the expression of E-cadherin got restored with downregulation of mesenchymal markers (Figure 4). The highest upregulation of E-cadherin was observed in the group treated with a combination of drugs followed by 2-ME (10 μM) and Qu (20 μM) treatment (Figure 4A). Similarly, in the case of mesenchymal markers, the highest downregulation was observed in the group treated with combination drugs followed by Qu and 2-ME treatment (N-cadherin) and 2-ME and Qu treatment (vimentin) (Figure 4B & C).

### Synergistic downregulation of cyclin D1 & β-catenin by Qu & 2-ME

**Figure 5.**
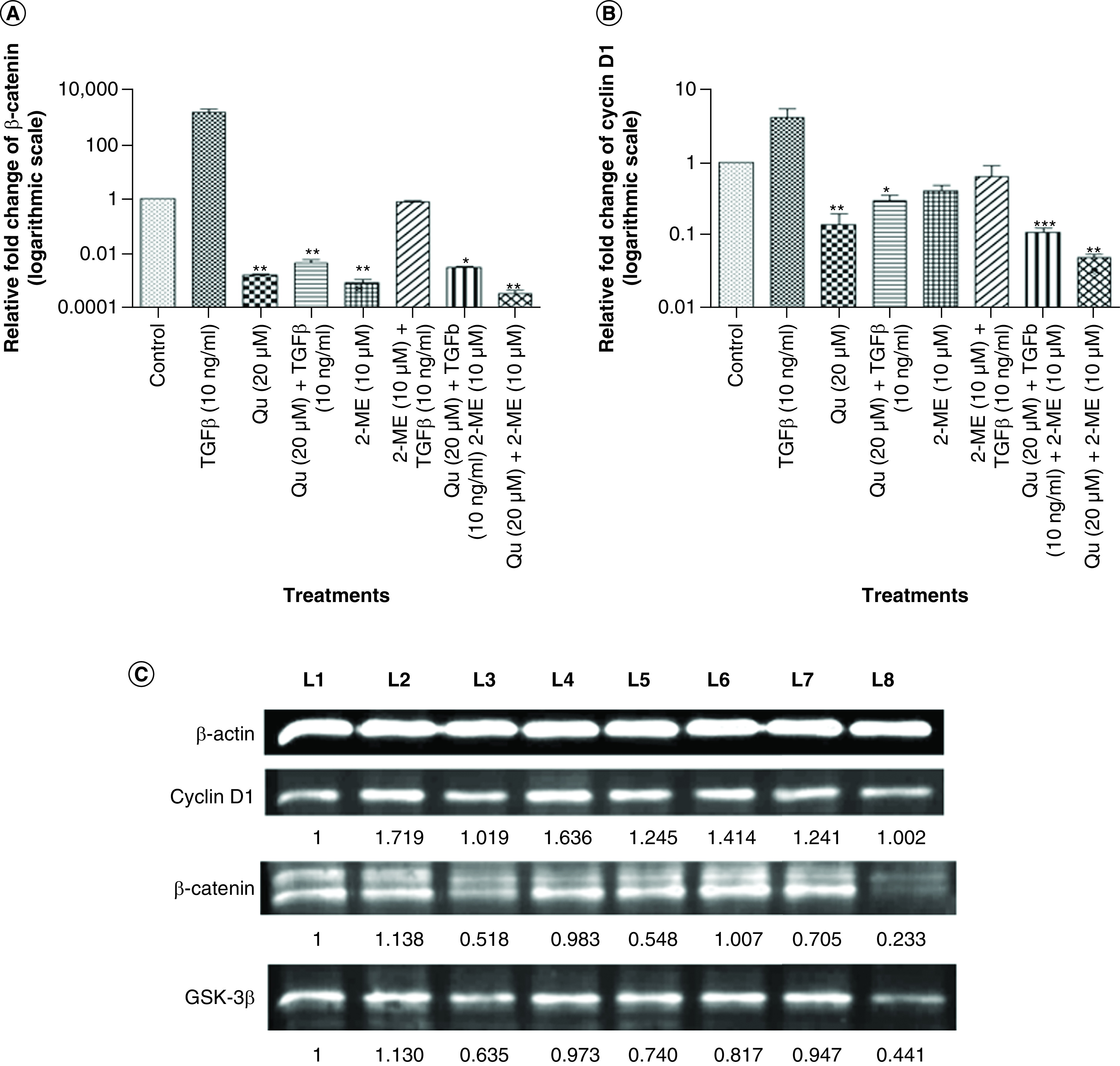
Quercetin and 2-methoxyestradiol downregulated Wnt signaling components of PC-3 cells at both gene and protein levels. Effect of all the three different groups of drugs on the mRNA expression of Wnt components: **(A)** β-catenin and **(B)** cyclin D1. Gene mRNA levels were normalized with β-actin and expressed as fold change with respect to the control group. Data represented as mean ± SEM from three independent experiments. The statistical analysis was performed using ANOVA with Tukey’s *post hoc* multicomparison test (*p < 0.05; **p < 0.01; ***p < 0.001). **(C)** The expression of Wnt signaling proteins *viz.*, cyclin D1 and β-catenin after treatment with different drug combinations was analyzed by western blotting. L1: Control; L2: TGF-β (20 ng/ml); L3: Quercetin (20 μM); L4: Quercetin (20 μM) + TGF-β (20 ng/ml); L5: 2-ME (10 μM); L6: 2-ME (10 μM) + TGF-β (20 ng/ml); L7: Quercetin (20 μM) + 2-ME (10 μM) + TGF-β (20 ng/ml); L8: Quercetin (20 μM) + 2-ME (10 μM). 2-ME: 2-methoxyestradiol; EMT: Epithelial–mesenchymal transition; Qu: Quercetin.

Cyclin D1 and β-catenin are Wnt signaling proteins that are reported to get accumulated in the nucleus upon Wnt signaling pathway activation. To study the effect of Qu and 2-ME on Wnt signaling proteins, expression level of cyclin D1 and β-catenin was analyzed by RT-PCR and western blotting, postdrug treatment. Treatment with TGF-β significantly upregulated the expression of cyclin D1 and β-catenin, whereas treatment with Qu (20 μM), 2-ME (10 μM) and Qu (20 μM) + 2-ME (10 μM) downregulated the expression of these markers at both gene (Figure 5A & B) and the protein level (Figure 5C). The most significant fold change was observed in the group treated with combination drug followed by Qu and 2-ME treatment (cyclin D1) and 2-ME and Qu treatment (β-catenin).

## Discussion

EMT is an orchestrated series of events in which epithelial cells gain mesenchymal properties with the simultaneous reorganization of the cytoskeletal to confer motility to the epithelial cells, thereby invading the 3D ECM with the initiation of a new transcriptional program to maintain the mesenchymal properties [29]. EMT can be both physiological and pathological. Physiological EMT occurs as a coordinated series of events, whereas physiological EMT occurs in an uncoordinated and cell-autonomous fashion [30]. Physiological EMT is involved in organogenesis, embryogenesis and wound healing, but pathological EMT is associated with disease conditions such as inflammation, fibrosis and tumor progression [31,32].

The process of EMT is known to play a pivotal role in developing metastatic castration-resistant PCa [33]. In PCa, several transcription factors are reported to induce EMT, such as Snail and Twist. Snail upregulation triggers EMT, which leads to downregulation of E-cadherin and upregulation of N-cadherin.

Gravdal *et al.* studied the significance of EMT in 104 men treated by radical prostatectomy. The tissue microarray and western blot analysis showed downregulation of E-cadherin and upregulation of N-cadherin. This E-cadherin to N-cadherin switch, which is suggestive of EMT is a strong indicator of clinical recurrence after radical prostatectomy [34]. Thus, their findings indicated that cell adhesion molecules could be used together with histological staining for not only prognosis of human PCa but also demonstrated the importance of EMT for the same.

In our previous work, we observed that Qu prevented TGF-β-induced expression of vimentin and N-cadherin and promoted E-cadherin expression in PC-3 cells, thus preventing TGF-β-induced EMT. Furthermore, it was observed that Qu significantly decreased the TGF-β-induced expression of Twist, Snail and Slug. Thus, our study reported that Qu could be a potential agent, which can be used to target and prevent EMT in PCa by regulating the components of the Wnt pathway [11]. Qu is reported to work synergistically with 2-ME in exerting anticancer effects in PCa cells [14]. However, the underlying molecular signaling pathway being targeted by these drugs in synergy is not well elucidated. Thus, we evaluated the synergistic effect of Qu and 2-ME in regulating TGF-β-induced EMT in PC-3 cells.

We evaluated the synergistic effect of Qu and 2-ME in PC-3 cells viability. Our data showed both Qu and 2-ME could inhibit PC-3 cell proliferation in a time-dependent manner; however, synergistically, these drugs showed a higher antiproliferative effect (Figure 1A). Also, the migratory potential of the PC-3 cells was evaluated upon drug treatment, individually and synergistically. The combination group (Qu [20 μM] + 2-ME [10 μM]) showed a higher antimigratory effect compared with the individual drug groups highlighting the interaction between Qu and 2-ME in exerting the antitumor properties toward PCa metastasis (Figure 1B & C). Additionally, Qu and 2-ME also affected the tumor-forming ability of PC-3 cells as depicted by the results of the soft agar colony-forming assay (Figure 1D), indicating the potential of Qu and 2-ME in impairing anchorage-independent growth of PC-3 cells.

Qu is reported to exert G2/M arrest in PC-3 cells [11,35], and 2-ME is shown to induce G2/M arrest with a simultaneous decline in G0/G1 population of cells in PCa [36]. To determine the synergistic effect of Qu and 2-ME on cell-cycle distribution of PC-3 cells, Flow cytometric analysis using PI staining was performed. Treatment with Qu and 2-ME showed an increase in the G0/G1 population of cells with a simultaneous decrease in G2/M population of cells. However, treatment with the combination group showed a decrease in both G0/G1 population as well as G2/M population of cells with an increase in sub-G0/G1 (apoptotic) population of cells (Figure 2A), indicating the synergistic effect Qu and 2-ME in inducing apoptosis of PC-3 cells. Similar findings have been reported in a hepatoma cell line where treatment with Qu and 2-ME induced apoptosis [37]. To further confirm the higher apoptotic potential of the combination group, caspase-3 activity assay was performed. Significant downregulation of caspase-3 activity of PC-3 cells was observed after 24 h of treatment with all the three groups (Figure 2B).

Castration-resistant prostate cancer (CRPC) followed by bone metastasis is the most aggressive form of PCa and often challenging to treat [38]. ALP is an enzyme expressed by bone and liver [39] and is one of the prognostic factors in determining bone metastasis in PCa [40]. To assess the effect of all the three groups of drug treatment on metastasis of PC-3 cells, ALP activity assay was performed. Inhibition of ALP was observed in all three groups with the highest inhibition in the group treated with 2-ME (Figure 3A). Knockdown of ALP is also reported to be associated with induction of MET (mesenchymal–epithelial transition) and cell death in PCa cells [41], indicating induction of MET upon drug treatment. Our finding that Qu and 2-ME can induce MET was further strengthened by upregulated expression of survivin mRNA expression, inhibitor of apoptosis family protein [26] upon treatment with all the three groups of drug and the highest inhibition was observed in the cells treated with the combination (Qu + 2-ME) treatment group (Figure 2C).

Flavonoids are reported to act as antioxidants and transfer hydrogen atoms to radicals, thereby limiting the negative effect of free radicals [18,42]. To assess the synergistic effect of Qu and 2-ME in exerting antioxidant properties on PC-3 cells, ROS activity of PC-3 cells was determined by Griess assay for all the three treatment groups (Figure 3B). The combination group (Qu and 2-ME) showed the highest % inhibition of NO radicals indicating the combination group exerting the highest antioxidant properties.

The process of EMT induction is marked by the downregulation of epithelial markers and upregulation of mesenchymal markers [43]. The EMT markers *viz.*, E-cadherin, N-cadherin and vimentin were studied by RT-PCR (Figure 4). In the present study, all the three groups of drugs could upregulate E-cadherin expression with simultaneous downregulation of mesenchymal markers. The combination group showed the highest inhibition of EMT by upregulation of E-cadherin and downregulation of N-cadherin and vimentin with approximately ten- and 100-fold, respectively. Very recently, a similar study has shown the synergistic effect of Qu with luteolin in inhibiting migratory and invasive potential of cervical cancer cells by regulating EMT [44].

Qu is reported as an inhibitor of the Wnt signaling pathway in many cancers such as colon cancer [38,44], leukemia cells and lymphoma [45]. In our previous study, we showed the inhibition of Wnt signaling components by Qu in PC-3 cells [10,11]. Loss of E-cadherin expression is associated with the accumulation of β-catenin in the cytoplasm and, finally, its translocation to the nucleus where it transcribes transcriptional repressors regulating EMT [4]. Also, β-catenin is a Wnt signaling component. Thus, the effect of drug treatment on Wnt signaling components, cyclin D1, β-catenin and GSK-3β was determined by RT-PCR and western blotting. In all the three treatment groups, downregulation of the expression of Wnt signaling components was observed both at the gene (Figure 5A & B) and at the protein level (Figure 5C), with the highest downregulation being observed in the combination group, in other words, with Qu and 2-ME.

## Conclusion

Collectively, we report that the molecular mechanisms driving Qu and 2-ME work synergistically to inhibit EMT via modulation of Wnt signaling components. Our results provide evidence that a combination of Qu and 2-ME may be an attractive alternative for the treatment of PCa.

## Future perspective

The Qu and 2-ME combination is a new clinical combination possessing potent antitumor activity on PCa by targeting the Wnt components. However, further experimental studies are required to unravel the exact mechanism of action to confirm the possibility of using these two anticancer drugs to treat PCa.

**Summary points**

- In this work, we demonstrated that a combination of quercetin (Qu) and 2-methoxyestradiol (2-Me) could inhibit epithelial–mesenchymal transition in prostate cancer (PCa) cells.
- Combination of Qu and 2-Me induced apoptosis and inhibited the proliferation of PCa cells.
- Combination of Qu and 2-Me impaired the migratory, tumor-forming ability and cell-cycle progression of PCa cells.
- Qu and 2-Me synergistically inhibited the alkaline phosphatase activity and promoted the reactive oxygen species activity of PCa cell.
- Wnt signaling pathway is identified as the key molecular player in driving the antitumorigenic effects of the combination of Qu and 2-Me in PCa.
- Our results provide important insights in understanding the mechanism of the anticancer effect of combination of Qu and 2-Me in regulating metastasis of PCa cells.
